# Effects of Miosis on Anterior Chamber Structure in Glaucoma Implant Surgery

**DOI:** 10.3390/jcm10051017

**Published:** 2021-03-02

**Authors:** Kee Sup Park, Kyoung Nam Kim, Jaeyoung Kim, Yeon Hee Lee, Sung Bok Lee, Chang-sik Kim

**Affiliations:** 1 Department of Ophthalmology, Chungnam National University College of Medicine, Daejeon 35015, Korea; red-mirr@hanmail.net (K.S.P.); scullism@gmail.com (J.K.); opticalyh@hanmail.net (Y.H.L.); sblee@cnu.ac.kr (S.B.L.); kcs61@cnu.ac.kr (C.-s.K.); 2 Department of Ophthalmology, Chungnam National University Hospital, Daejeon 35015, Korea

**Keywords:** pilocarpine, miosis, glaucoma implant surgery, anterior chamber

## Abstract

We investigated changes in anterior chamber (AC) structure after miosis in phakic eyes and pseudophakic eyes with glaucoma. In this prospective study, patients scheduled for glaucoma implant surgery were examined using anterior segment optical coherence tomography before and after miosis. Four AC parameters (AC angle, peripheral anterior chamber (PAC) depth, central anterior chamber (CAC) depth, and AC area) were analyzed before and after miosis, and then compared between phakic and pseudophakic eyes. Twenty-nine phakic eyes and 36 pseudophakic eyes were enrolled. The AC angle widened after miosis in both the phakia and pseudophakia groups (*p* = 0.019 and *p* < 0.001, respectively). In the phakia group, CAC depth (*p* < 0.001) and AC area (*p* = 0.02) were significantly reduced after miosis, and the reductions in PAC depth, CAC depth, and AC area were significantly greater than in the pseudophakia group (all *p* < 0.05). Twenty-five patients (86.2%) in the phakia group and 17 (47.2%) in the pseudophakia group had reduced CAC depth (*p* = 0.004). Although miosis increased the AC angle in both groups, AC depth decreased in most phakic eyes and a substantial number of pseudophakic eyes. Preoperative miosis before glaucoma implant surgery may interfere with implant tube placement distant from the cornea during insertion into the AC.

## 1. Introduction

The instillation of pilocarpine for the purpose of miosis may be performed before various ophthalmic surgeries or laser treatments. For example, miosis is helpful to thinly stretch the peripheral iris during peripheral laser iridotomy, prevent excessive iris resection during trabeculectomy, and prevent damage to the lens due to anterior chamber (AC) puncture and tube insertion during glaucoma implant surgery [1,2,3,4].

Glaucoma implant surgery is a common glaucoma treatment procedure. The reported surgical success rates and risks of complications are comparable with those of trabeculectomy, which is the most frequent procedure [5,6,7]. Although the surgical results of glaucoma implant surgery are comparable with those of trabeculectomy, concerns remain about progressive corneal endothelial damage, subsequent corneal decompensation, and resultant vision loss [3,4,8,9]. Recently, several studies using anterior segment optical coherence tomography (AS-OCT) have reported the effects on the corneal endothelium of tube insertion in the AC during glaucoma implant surgery. Koo et al. [10] and Tan et al. [11] concluded that tubes positioned closer to the cornea led to increased damage to adjacent corneal endothelium. Lee et al. reported that the angle between the tube and the cornea was narrower in eyes with significant corneal endothelial damage than in eyes without damage (28.67 ± 7.79° vs. 36.35 ± 5.35°, *p* < 0.001), and the distance between the tube and the cornea was shorter in eyes with corneal endothelial damage than in eyes without damage (0.98 ± 0.38 mm vs. 1.26 ± 0.39 mm, *p* = 0.002) [12]. They suggested that during its insertion into the AC, a considerable distance is required between the tube and the cornea to reduce corneal endothelial damage after glaucoma implant surgery [10,11,12].

In phakic eyes, pilocarpine-induced miosis reduces the AC depth in association with a reduction in AC volume and in the radius of lens curvature [13,14]. Conversely, the effects of pilocarpine in pseudophakic eyes are unclear; previous studies have yielded conflicting results [15,16,17,18,19]. Lea et al. [18] reported a forward shift of a rigid intraocular lens (IOL, a maximum of 0.25 mm), but found no significant change in a flexible IOL as measured by optical pachymetry. In contrast, Findl et al. [19] observed either no significant shift or a slight backward shift when using conventional three-piece IOLs, as measured by partial coherence interferometry.

Determination of the effects of preoperative miosis on AC structure, depending on the presence of phakia or pseudophakia, will help clarify whether miosis is helpful in tube placement during glaucoma implant surgery. To the best of our knowledge, no study has directly compared changes in the AC after the instillation of pilocarpine in phakic and pseudophakic eyes. Therefore, we compared changes in AC structure between phakic and pseudophakic eyes using AS-OCT after pilocarpine-induced miosis in patients scheduled for glaucoma implant surgery. Additionally, to evaluate the AC structure in relation to tube insertion, we measured the peripheral and center AC depths based on the iris surface, unlike previous studies that measured only central anterior chamber (CAC) depth based on the lens surface.

## 2. Methods

### 2.1. Patients

This prospective study was approved by the Institutional Review Board of Chungnam National University Hospital in the Republic of Korea (IRB number: 2018-03-040). It was conducted in accordance with all relevant requirements of the Declaration of Helsinki. Informed consent was acquired from all participants. Patients who were scheduled for Ahmed glaucoma valve implantation in our glaucoma clinic between June 2018 and January 2020 were consecutively enrolled in the study. Neovascular glaucoma, secondary glaucoma resulting from uveitis, ocular surgery, and glaucoma with a wide conjunctival scar from previous ocular surgery such as trabeculectomy were included as indications for Ahmed glaucoma valve implantation.

Patients were excluded if they had considerable corneal opacity that interfered with AS-OCT imaging, a fixed pupil due to iris sphincter injury or posterior synechiae, a history of acute angle closure attack, and/or evidence of zonular weakness (e.g., phacodonesis, pseudophacodonesis, and vitreous strands in the AC). Patients were also excluded if they had undergone intraocular surgery within the preceding 3 months. In each patient, the eye that met the inclusion criteria was selected. When both eyes met the criteria, one eye was selected for analysis using the random sampling method in Microsoft Excel^®^ (Microsoft Corp., Redmond, WA, USA).

All enrolled patients underwent complete ophthalmic examinations, including best-corrected visual acuity, auto-refractometry, slit-lamp biomicroscopy, Goldmann applanation tonometry, gonioscopy, dilated fundus examination, and pachymetry (Ocuscan RxP; Alcon, Fort Worth, TX, USA), as well as measurements of axial length, AC depth, and keratometry (IOLMaster, Carl Zeiss, Jena, Germany).

### 2.2. Anterior Segment Optical Coherence Tomography (AS-OCT)

AS-OCT was performed before and after miosis. All patients underwent AS-OCT (Cirrus HD OCT; Carl Zeiss Meditec, Dublin, CA, USA) at the last outpatient clinic visit before glaucoma surgery. AS-OCT was performed without miosis. Subsequently, 2% pilocarpine (Alcon) was instilled twice at 5-min intervals to induce miosis. AS-OCT was repeated 50 min later. During these examinations, the patients were asked to fixate on an internal target. Images without artifacts due to eye motion and blinking were used for analysis. The scanning axis was aligned with the horizontal line passing through the center of the pupil.

**Figure 1:**
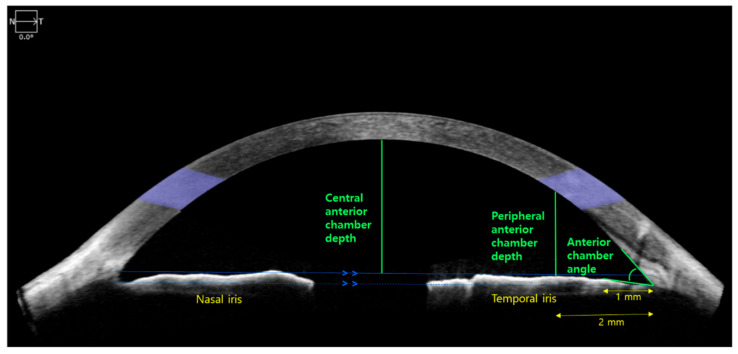
Anterior segment optical coherence tomography (AS-OCT) parameters. Among the four anterior chamber (AC) parameters used in this study, central anterior chamber (CAC) depth, peripheral anterior chamber (PAC) depth, and AC angle were measured using an integrated ruler and angle indicator. For the AC area, the value was measured automatically by AS-OCT.

Four AC parameters were acquired by AS-OCT (Figure 1). The first three parameters were measured using an integrated ruler and angle indicator: (1) AC angle (°) was defined as the angle between the corneal endothelium and anterior surface of the iris, 1 mm from the vertex of AC recess; (2) CAC depth (mm) was measured as the deepest vertical distance to the corneal endothelium following the construction of a line parallel to the horizontal line connecting the nasal to temporal scleral spur and in contact with the anterior surface of the temporal iris; and (3) peripheral anterior chamber depth (PAC depth, mm) was defined as the vertical distance from the iris surface, 2 mm from the vertex of AC recess to the corneal endothelium. Both AC angle and PAC depth were acquired from the temporal side. The fourth parameter, AC area (mm^2^), was measured automatically with built-in software.

### 2.3. Statistical Analysis

PASW software, version 18.0 (SPSS Inc., Chicago, IL, USA) was used for all statistical analyses. Enrolled patients were divided into two groups depending on their lens status (phakic or pseudophakic). Demographic characteristics were compared between the two groups using Student’s *t*-test and Fisher’s exact test. Student’s *t*-test was used to compare AC parameters between the two groups. Paired *t*-tests were used to analyze post-miosis changes in AC parameters in each group, and correlations between AC parameters before and after miosis were analyzed. Differences in subgroup composition according to changes in AC parameters between two groups were analyzed using the Pearson chi-squared test. In all analyses, *p* < 0.05 was considered to indicate statistical significance.

## 3. Results

Data from 65 eyes of 65 patients scheduled for glaucoma implant surgery were analyzed. These included 29 phakic eyes and 36 pseudophakic eyes. The mean ages of patients in the phakia and pseudophakia groups were 62.1 ± 12.9 years and 64.8 ± 13.9 years, respectively (*p* = 0.419). The mean AC depth measured by IOLMaster, from the corneal endothelium to the anterior surface of the lens, was shallower in the phakia group than in the pseudophakia group (3.0 ± 0.6 mm vs. 4.3 ± 0.7 mm, *p* < 0.001). The mean axial lengths did not differ significantly between groups (23.4 ± 1.4 mm vs. 23.8 ± 1.2 mm, *p* = 0.180, Table 1).

Table 2 compares AC parameters measured by AS-OCT between the phakia and pseudophakia groups. All AC parameters before and after miosis were significantly smaller in the phakia group (all *p* < 0.001). In the phakia group, the AC angle was wider after miosis than before miosis (△1.48 ± 3.21°, *p* = 0.019). Post-miotic CAC depth (△−0.12 ± 0.11 mm, *p* < 0.001) and AC area (△−0.88 ± 1.38 mm^2^, *p* = 0.02) were significantly lower than their corresponding pre-miotic values. In the pseudophakia group, AC angle was the only parameter that significantly increased after miosis (△1.64 ± 2.39°, *p* < 0.001). Differences between pre- and post-miosis PAC depth, CAC depth, and AC area values were larger in the phakia group than in the pseudophakia group (*p* = 0.043, *p* < 0.001, and *p* = 0.036, respectively).

**Figure 2:**
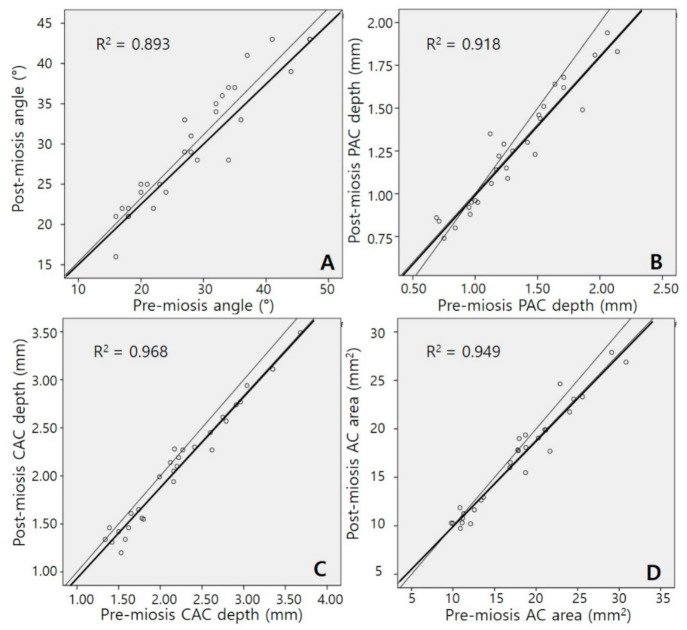
Scatter plots of anterior chamber (AC) parameters in the phakia group. (**A**) AC angle, (**B**) peripheral anterior chamber (PAC) depth, (**C**) central anterior chamber (CAC) depth, and (**D**) AC area. Correlations between pre-miosis and post-miosis values in all parameters were strongly positive and statistically significant (all *p* < 0.001). The thin line represents Y = X.

**Figure 3:**
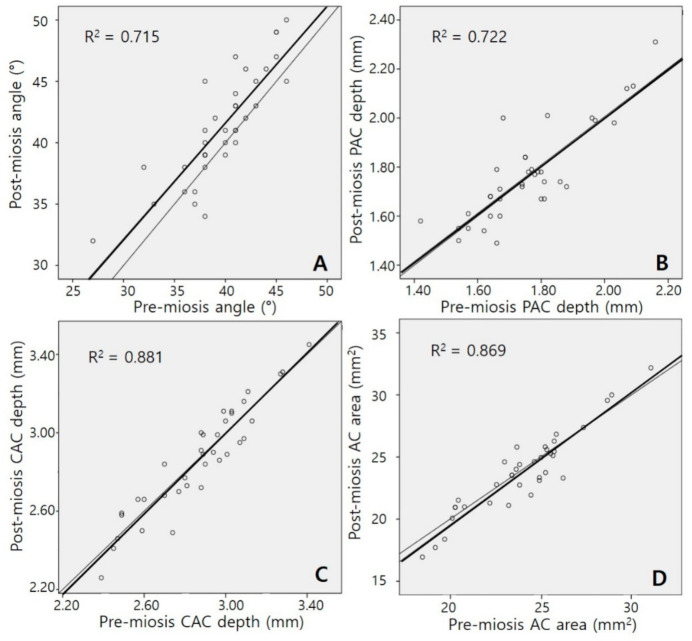
Scatter plots of anterior chamber (AC) parameters in the pseudophakia group. (**A**) AC angle, (**B**) peripheral anterior chamber (PAC) depth, (**C**) central anterior chamber (CAC) depth, and (**D**) AC area. Correlations between pre-miosis and post-miosis values in all parameters were positive and statistically significant (all *p* < 0.001). The thin line represents Y = X.

Figure 2 and Figure 3 present the relationships between pre-miosis and post-miosis AC parameters in the phakia and pseudophakia groups, respectively. In both groups, strong positive correlations were observed in all four parameters between pre-miosis and post-miosis values (all *p* < 0.001). Correlation coefficients of the AC angle, PAC depth, and CAC depth were lower in the pseudophakia group than in the phakia group (Fisher’s Z transformation, z = 2.076, z = 2.535, and z = 2.627; *p* = 0.038, *p* = 0.011, and *p* = 0.009, respectively). The correlation coefficient of the AC area showed a trend, but the result was not statistically significant (z = 1.874, *p* = 0.061).

Table 3 lists the distribution of patients stratified according to changes in AC parameters after miosis. In both groups, most patients had an enhancement of AC angle (*p* = 0.678). Most patients in the phakia group had reductions in PAC depth, CAC depth, and AC area, whereas approximately equal numbers of patients in the pseudophakia group had reductions and enhancements of those parameters (*p* = 0.019, *p* = 0.002, and *p* = 0.019, respectively).

**Figure 4:**
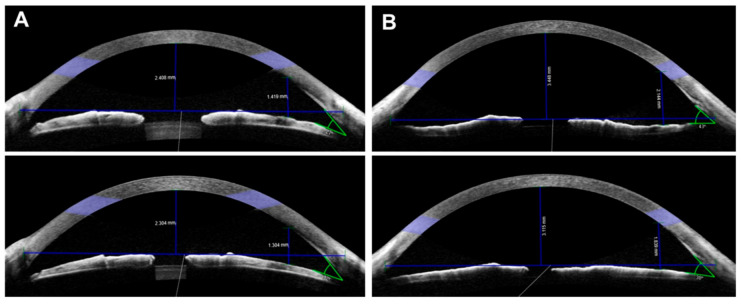
Anterior segment optical coherence tomography (AS-OCT) images of representative patients in the phakia group. Top depicts pre-miotic image and bottom depicts post-miotic image. (**A**) Anterior chamber (AC) angle widened (27° to 32°), but central (CAC) and peripheral anterior chamber (PAC) depths became shallower after miosis (2.408 mm to 2.304 mm and 1.419 mm to 1.304 mm, respectively). (**B**) AC angle (43° to 36°) narrowed and CAC and PAC depths (3.448 to 3.115 mm and 2.144 to 1.839 mm, respectively) became shallower after miosis.

Table 4 lists the distribution of the patients stratified according to the combination of changes in AC angle and CAC depth. In the phakia group, 19 patients (65.5%) had an enhanced AC angle and a reduced CAC depth. Six patients (20.7%) had a reduced AC angle and a reduced CAC depth. Figure 4 shows representative patients from these two subgroups (A and B, respectively). In the pseudophakia group, 19 patients (52.8%) had an enhanced AC angle and an enhanced CAC depth, and 11 patients (30.6%) had an enhanced AC angle and a reduced CAC depth (*p* = 0.004).

## 4. Discussion

We found that post-miosis changes in AC structure differed between phakic and pseudophakic eyes. In phakic eyes, most (86.2%) had a shallower AC depth after miosis, regardless of the change in AC angle. In pseudophakic eyes, the change in AC structure was relatively inconsistent. In 52.8% of patients, the AC depth became deeper and the AC angle became wider, and in 47.2% of patients, the AC depth became shallower regardless of the change in AC angle. Therefore, in considerable numbers of patients with phakia or pseudophakia, the use of pilocarpine prior to glaucoma implant surgery could adversely affect implant tube placement distant from the cornea during insertion into the AC.

Glaucoma implant surgery is increasingly being performed as an alternative or equivalent choice to trabeculectomy [5,7,20]. However, some complications such as hypotony, shallow AC, hyphema, tube–corneal contact, choroidal effusion, cataract formation, endophthalmitis, and corneal endothelial damage may result from glaucoma implant surgery [9]. Of these, progressive corneal endothelial cell loss is a major long-term complication, which leads to corneal decompensation [6,10,21]. The mechanism of corneal endothelial cell damage after glaucoma implant surgery has not been fully elucidated, but several hypotheses have been suggested. One of the most promising hypotheses is that tube insertion in the AC will affect corneal endothelial damage [9]. Some studies have yielded results to support this hypothesis. Kim et al. [22] reported that the corneal endothelial cell densities were reduced by 22.4%, 28.7%, 19.4%, and 18.3% at superior, supratemporal, supranasal, and central locations after glaucoma implant surgery. Similarly, Lee et al. [23] reported corneal endothelial cell losses of 20.3%, 22.6%, 18.1%, and 15.4% at superior, supratemporal, supranasal, and central locations, respectively. In both studies, the greatest cell loss was evident in the supratemporal location, where the tube was located. In studies using AS imaging [10,11,12], a shorter distance and a narrower angle between the tube and corneal endothelium were both risk factors for corneal endothelial cell loss. These results suggest that it is preferable to place the tube distant from the cornea during insertion in the AC, thereby reducing corneal endothelial damage after glaucoma implant surgery. Park et al. [24] reported that the tube in the AC gradually moved toward the corneal endothelium after glaucoma implant surgery. Therefore, it is also important to position the tube in the posterior portion of the AC to prevent long-term corneal damage after glaucoma implant surgery.

Some surgical techniques have been introduced to reduce corneal endothelial damage. Pars plana tube insertion has been performed in combination with penetrating keratoplasty when traditional AC tube insertion is not possible. However, pars plana tube insertion is only feasible in vitrectomized eyes [25,26,27]. Although ciliary sulcus tube insertion does not require vitrectomy, it can be used in pseudophakic eyes to avoid lens damage [28,29]. In general, during glaucoma implant surgery, the tube is inserted into the AC distant from the corneal endothelium and parallel to the front surface of the iris. Approved indications for the ophthalmic use of pilocarpine include a reduction in elevated intraocular pressure in patients with glaucoma or ocular hypertension, the prevention of postoperative elevated intraocular pressure, and the induction of miosis [30]. No consensus has been reached in terms of whether pilocarpine should be used before glaucoma implant surgery. Therefore, miosis is selectively performed in accordance with the operator’s preferences. Preoperative miosis is expected to help prevent damage to the lens during AC puncture or tube insertion during surgery. However, we found that in phakic eyes, shallower AC depth is likely to hinder the insertion of the tube distant from the cornea. During tube insertion, to prevent iris incarceration into the tube opening, the tube must be inserted in parallel without contacting the iris. If the tube is inserted in a shallow AC, it will be inserted nearer to the cornea.

The administration of pilocarpine causes iris sphincter contraction, along with the contraction of the ciliary muscle, which leads to zonule relaxation and changes in both lens shape and thickness. This series of changes moves the lens-iris diaphragm forward. Therefore, the AC depth is slightly reduced [15,16,17]. In our study, the correlation coefficients of pre-miosis and post-miosis AC parameters were lower in pseudophakic eyes than in phakic eyes, presumably because the lens-iris diaphragm is less affected by ciliary muscle contraction in pseudophakic eyes [19]. Previous studies have demonstrated differences in post-miosis AC depth changes in pseudophakic eyes, depending on the IOL shape and material [17,18,19].

Rękas et al. [31] studied changes in AC configuration after cataract surgery and classified subjects into four groups based on preoperative parameters, such as the AC depth, AC angle, lens thickness, and axial length. The AC configuration was significantly different among the groups. Although these preoperative parameters are related to the amount of change in the AC after cataract surgery [32,33,34], Rękas et al. showed that the changes in AC depth and angle after cataract surgery explained only 42.65% of the change predicted by the preoperative parameters. Altan et al. [35] and Huang et al. [36] suggested a possible influence of other anatomical structures and postoperative changes in the eye, such as ciliary processes, ciliary zonule, and the iris. In our study, it was expected that various anatomical factors would affect the AC after miosis induced by pilocarpine instillation. However, in clinical practice, the most useful way to determine whether miosis is advantageous when preparing for glaucoma implant surgery is to directly compare the AC before and after miosis.

Considering the potential for bias, we excluded patients with zonular weakness, which is known to cause considerable change in AC depth due to miosis or mydriasis [23,24,25]. Importantly, patients with zonular weakness who are scheduled for surgery may have a larger reduction in AC depth than the values confirmed in this study.

This study had some limitations. First, we could not analyze whether changes in AC structure after miosis differed among subgroups according to glaucoma type because relatively few patients were enrolled. To test this hypothesis, additional studies are needed with larger numbers of patients. Second, we could not compare actual tube positions between insertion performed with and without miosis because we did not perform miosis during glaucoma implant surgery in patients if the AC angle became narrow and/or the AC depth became shallow after miosis.

In conclusion, when miosis is performed during preparation for glaucoma implant surgery, patients with phakic eyes may have a shallow AC depth that causes difficulty in positioning the tube distant from the cornea. In some patients with a considerable reduction in AC depth after miosis, cataract surgery may be considered to avoid tube insertion near the cornea. Additionally, in many pseudophakic eyes, the AC may become shallow after miosis. Therefore, if miosis is performed routinely before glaucoma implant surgery, the suitability of miosis in each patient should be checked before surgery by examining changes in the AC due to miosis.

## Figures and Tables

**Table 1:** Demographic characteristics of patients.

	Phakia Group (*n* = 29)	Pseudophakia Group (*n* = 36)	*p*-Value *
Age (years)	62.1 ± 12.9	64.8 ± 13.9	0.419
Sex (male/female)	13/16	20/16	0.459 †
Diabetes mellitus (*n*)	8 (27.6%)	17 (47.2%)	0.129 †
Hypertension (*n*)	10 (34.5%)	16 (44.4%)	0.455 †
Type of glaucoma (*n*)			
Primary open-angle	11	14	
Primary angle-closure	7	5	
Uveitic	3	4	
Neovascular	5	9	
Pseudoexfoliation	3	4	
Axial length (mm)	23.4 ± 1.4	23.8 ± 1.2	0.180
Anterior chamber depth (mm)	3.0 ± 0.6	4.3 ± 0.7	<0.001
Keratometry (diopter)	44.7 ± 1.8	44.1 ± 1.5	0.131
Central corneal thickness (um)	536.5 ± 30.1	538.8 ± 24.3	0.729
Spherical equivalent (diopter)	−0.6 ± 3.6	−0.9 ± 1.2	0.695

Data are shown as mean ± standard deviation or *n*, * Student’s *t*-test, † Fisher’s exact test.

**Table 2:** Comparisons of anterior chamber parameters between phakia and pseudophakia groups before and after miosis.

	Phakia Group (*n* = 29)	Pseudophakia Group (*n* = 36)	*p*-Value *
Pre-miosis			
Anterior chamber angle (°)	28.4 ± 9.3	39.7 ± 4.0	<0.001
Peripheral anterior chamber depth (mm)	1.31 ± 0.40	1.76 ± 0.17	<0.001
Central anterior chamber depth (mm)	2.20 ± 0.62	2.86 ± 0.25	<0.001
Anterior chamber area (mm^2^)	17.63 ± 5.87	23.97 ± 2.83	<0.001
Post-miosis			
Anterior chamber angle (°)	29.90 ± 7.77	41.33 ± 4.46	<0.001
Peripheral anterior chamber depth (mm)	1.26 ± 0.34	1.77 ± 0.19	<0.001
Central anterior chamber depth (mm)	2.07 ± 0.60	2.86 ± 0.27	<0.001
Anterior chamber area (mm^2^)	16.76 ± 5.36	23.78 ± 3.24	<0.001
Differences †			
Anterior chamber angle (°)	1.48 ± 3.21 (0.019)	1.64 ± 2.39 (<0.001)	0.823
Peripheral anterior chamber depth (mm)	−0.06 ± 0.13 (0.571)	−0.003 ± 0.089 (0.590)	0.043
Central anterior chamber depth (mm)	−0.12 ± 0.11 (<0.001)	−0.004 ± 0.095 (0.784)	<0.001
Anterior chamber area (mm^2^)	−0.88 ± 1.38 (0.02)	−0.19 ± 1.19 (0.335)	0.036

Data are shown as mean ± standard deviation, * Paired *t*-test, † Values indicate post-miosis−pre-miosis.

**Table 3:** Distribution of patients stratified according to changes in anterior chamber structure after miosis.

	Enhancement	Reduction	*p*-Value
Anterior chamber angle			
Phakia group (*n*)	23	6	0.678
Pseudophakia group (*n*)	30	6	
Peripheral anterior chamber depth			
Phakia group (*n*)	7	22	0.019
Pseudophakia group (*n*)	19	17	
Central anterior chamber depth			
Phakia group (*n*)	4	25	0.002
Pseudophakia group (*n*)	19	17	
Anterior chamber area			
Phakia group (*n*)	7	22	0.019
Pseudophakia group (*n*)	19	17	

Pearson chi-squared test.

**Table 4:** Distribution of patients stratified according to the combination of changes in anterior chamber angle and central anterior chamber depth after miosis.

		Phakia Group (*n* = 29)		Pseudophakia Group (*n* = 36)	
		Anterior Chamber Angle		Anterior Chamber Angle	
		Enhancement	Reduction	Enhancement	Reduction
Central anterior chamber depth	Enhancement	4 (13.8%)	0	19 (52.8%)	0
	Reduction	19 (65.5%)	6 (20.7%)	11 (30.6%)	6 (16.6%)
*p*-value		0.004			

Pearson chi-squared test.

## Data Availability

The data presented in this study are available on request from the corresponding author. The data are not publicly available due to privacy and ethical reasons.
